# Draft Genome Sequence of Clinical Isolate USM039K of the Pathogenic Yeast Candida parapsilosis

**DOI:** 10.1128/mra.00841-22

**Published:** 2022-10-27

**Authors:** Dina Yamin, Wan Khairunnisa Wan Juhari, NurWaliyuddin Hanis Zainal Abidin, Shuhaila Mat-Sharani, Azian Harun

**Affiliations:** a Department of Medical Microbiology and Parasitology, School of Medical Sciences, Universiti Sains Malaysia, Kubang Kerian, Kelantan, Malaysia; b Malaysian Node of the Human Variome Project, School of Medical Sciences, Universiti Sains Malaysia, Kubang Kerian, Kelantan, Malaysia; c Human Identification/DNA Unit, School of Health Sciences, Universiti Sains Malaysia, Kubang Kerian, Kelantan, Malaysia; d Biomedicine Program, School of Health Sciences, Universiti Sains Malaysia, Kubang Kerian, Kelantan, Malaysia; e Hospital Universiti Sains Malaysia, Kota Bharu, Kelantan, Malaysia; University of California, Riverside

## Abstract

Here, we announce the draft genome sequence of a Candida parapsilosis clinical isolate (USM039K) recovered from a patient with catheter-related bloodstream infection (CRBSI). The genome size is 12,860,016 bp long, with 188 scaffolds, a G+C content of 38.65%, and 5,467 genes.

## ANNOUNCEMENT

This report is to present the draft genome sequence of a clinical isolate of Candida parapsilosis. C. parapsilosis is a yeast classified under cellular organisms, superkingdom of Eukaryota, kingdom of Fungi, subkingdom of Dikarya, phylum of Ascomycota, subfamily of Saccharomycotina, class of Saccharomycetes, order of Saccharomycetales, family of Debaryomycetaceae, and genus of *Candida*. This species is known as a causative agent for candidemia (1). The draft genome sequence of this species will enhance the current knowledge of the genetic variation among C. parapsilosis isolates. The organism was isolated from a patient with catheter-related bloodstream infection (CRBSI) in Hospital Universiti Sains Malaysia (USM), Kelantan, Malaysia. Ethical approvals were obtained from the Human Research Ethics of Universiti Sains Malaysia (JEPeM-USM-16040162).

The isolate was cultivated overnight on a Sabouraud dextrose agar plate with yeast cultivation medium and incubated at 37°C. Pure colonies were collected during the stationary phase. DNA extraction was done using phenol-chloroform extraction followed by ethanol precipitation (2). Species identity was confirmed by the sequences of the internal transcribed spacer (ITS) region of ribosomal DNA (3). Sample quantity and quality were assessed prior to sequencing using the Illumina NovaSeq6000 system.

Briefly, DNA fragmentation was performed by sonication to 350 bp. These fragments were end polished, A tailed, and ligated with the Illumina sequencing adapter prior to amplification by PCR. Library preparation was accomplished for the qualified DNA using the NEBNext Ultra DNA library prep kit (New England BioLabs [NEB], USA) according to the manufacturer’s protocol. DNA libraries were purified using the AMPure XP system, analyzed for size distribution using an Agilent 2100 bioanalyzer, and quantified by real-time PCR. Then, the qualified libraries were sequenced to an average sequencing depth of 250× using the Illumina NovaSeq 150PE protocol generating 2 × 150-bp paired-end reads. The raw output was transformed into raw reads by CASAVA base calling and converted into FASTQ file format, which produced 22,252,138 raw paired-end reads. The quality of the FASTQ file was assessed using FastQC software. The adapter and low-quality sequences were discarded using Trimmomatic (v0.36) by performing sliding window trimming with a minimum average quality of 20 and a minimum sequence read length of 20 bases (4).

*De novo* genome assembly for the C. parapsilosis USM039K strain and annotation analyses were conducted using the Galaxy platform with default parameters or those otherwise stated (5). The assembly was generated by SPAdes (v3.12.0) (6), and the quality was assessed using Quality Assessment Tool (QUAST) (v5.0.2) (7). The draft genome resulted in a total of 188 scaffolds (≥1,000 bp) with a total length of 12,860,016 bp, a G+C content of 38.65%, an *N*_50_ value of 173,876 bp, and an *L*_50_ value of 23. The longest scaffold in the draft genome is 533,946 bp. The assemblies produced 98% of aligned sequences with the reference genome (CDC317; GCA_000182765.2). Interspersed and low-complexity repetitive sequences were masked using RepeatMasker v4.0.9 (http://repeatmasker.org) with combined Dfam_3.0 and RepBase-20181026 databases according to the fungal species. The results showed that 287,166 bp (2.23%) was masked (8). The gene prediction of final scaffolds was performed using MAKER (v2.31.10) (9), AUGUSTUS v3.3.3 (10), and SNAP software (11). The species training was Candida albicans. In total, 5,467 genes were predicted, consisting of 4,655 (83.5%) single-exon genes and 923 (16.5%) multiexon genes in this newly sequenced strain.

### Data availability.

This whole-genome shotgun project has been deposited and is available at DDBJ/ENA/GenBank (BioProject number PRJNA610714 and assembly accession number JADCQT000000000). The version described in this paper is the first version, JADCQT010000000. Raw reads are available in the NCBI Sequence Read Archive under accession number SRR11249109.
